# The Explanation of Adolescent Delinquent Behaviors Based on Jessor's Problem Behavior Theory (PBT) in Iran: The Role of Individual Vulnerability, Opportunity Risk Availability, and Perceived Support

**DOI:** 10.3389/fpsyt.2022.744794

**Published:** 2022-01-28

**Authors:** Mona Darvishi, Mohammad Kazem Atef Vahid, Mojtaba Elhami Athar, Elizabeth Trejos-Castillo, Mojtaba Habibi Asgarabad

**Affiliations:** ^1^Department of Health Psychology, School of Behavioral Sciences and Mental Health (Tehran Institute of Psychiatry), Iran University of Medical Sciences, Tehran, Iran; ^2^Department of Clinical Psychology, School of Behavioral Sciences and Mental Health (Tehran Institute of Psychiatry), Iran University of Medical Sciences, Tehran, Iran; ^3^Department of Human Development and Family Sciences, Texas Tech University, Lubbock, TX, United States; ^4^Health Promotion Research Center, Iran University of Medical Sciences, Tehran, Iran; ^5^Department of Psychology, Norwegian University of Science and Technology, Trondheim, Norway; ^6^Center of Excellence in Cognitive Neuropsychology, Institute for Cognitive and Brain Sciences, Shahid Beheshti University, Tehran, Iran

**Keywords:** delinquent behavior, individual vulnerability, risk availability, problem behavior theory, adolescence, support

## Abstract

This study tested the generality of Problem Behavior Theory (PBT) in explaining adolescents' problem behavior in Iran. Data were collected from 392 adolescents (M_age_ = 15.97, *SD* = 1.12, 55.4% girls) who completed the Adolescent Health and Development Questionnaire (AHDQ) to assess the individual vulnerability, opportunity risk availability, perceived support, and delinquent behaviors. Results indicated that individual vulnerability and opportunity risk availability had a significant relationship with delinquent behaviors and a significant interaction with perceived support in their influence on delinquent behaviors. Further, perceived support was negatively associated with delinquent behaviors. Our results were consistent with PBT's explanatory model for adolescents' problem behavior in Western countries and are informative about problem behavior involvement among Iranian adolescents and the design of interventions.

## Introduction

Delinquent behavior refers to an action the commitment of which confronts the offender to the laws of the civil society (1). Some of the delinquent behaviors include theft, property damage, physical aggression, selling drugs, burglary, robbery, vandalism, and avoiding school (2), which could lead to poor educational performance, school absenteeism (3), escape from home (4), substance use, depression/anxiety, self-harm (5), and even increased probability of unnatural death caused by suicide, murder, and alcohol abuse (6, 7). Even though the problem behaviors rate has declined in some countries (e.g., the United States) (8), it is still among the widespread risk behaviors in many societies (9–11).

A broad range of behavioral problems such as antisocial behavior (12), drug use (13), and alcohol consumption (14) appears during adolescents. In the same vein, some records show that engagement in antisocial behavior comes to a climax from mid-to-late- adolescence' years (15). For example, 1,154,096 youths were put in jail in the US in 2010 (15). In addition, a study indicated that among 50 percent of twelfth-grade students had used illegal substances during their lifespan, 70% had drunk alcohol, and 19% smoked currently (14). Further, several studies examined problem behaviors among adolescents in Iran, which were primarily epidemiological [e.g., (16–18)]. For instance, Rashid (18) studied the prevalence of problem behaviors among adolescents in Iran; results indicated that hookah smoking (51.5%), cigarette smoking (35.2%), beating outside the house (except school) (28.1%), and drinking (27.4%) were respectively the most common problem behaviors among adolescents. A high frequency of engagement in problem behavior may lead to some inconvenience. For example, adolescents involved in problem behaviors are more likely to have problems in different areas, such as lower psychosocial adaptation or physical health, poorer life expectations, and a difficult transition into adulthood (19). Adolescent problem behaviors may also result in long-term developmental problems, continuing to adulthood, though these behaviors may not become chronic for most of them (20).

Several theories have been developed to explain delinquency in adolescents, including General Strain Theory (21, 22), Object Relations Theory (23), Interactional Theory (24), Developmental Theory (25), Social Control Theory (26, 27), Social Learning Theory (28), Social Development Model (29), and Problem Behavior Theory (30).

**Figure 1:**
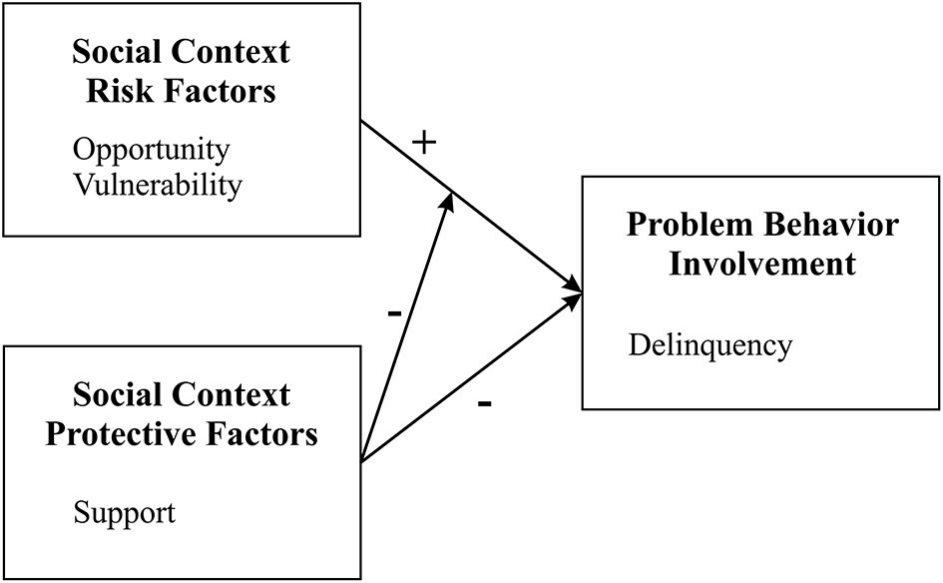
The protection-risk model of social context and adolescent problem behavior involvement (33). The “+” and “-” signs indicate a positive or negative impact on problem behavior involvement.

PBT that is the main focus of the current study, is one of the most comprehensive theories regarding the etiology of problem behaviors. PBT is a social–psychological framework that explains the association between psychosocial protective and risk factors and involvement in problem behaviors (e.g., delinquent behavior, substance use, problem drinking, and early sexual intercourse) (31). According to PBT, problem behaviors result from the interaction between risk factors such as opportunity risk availability (e.g., exposure to risk opportunities, such as gang membership) and individual vulnerability (e.g., individual-level features, such as stress, depression, low self-esteem, or perceived hindered access to the achievement of a prosperous life) and protective factors (e.g., the support given by friends, teachers, and neighbors) (32). While risk factors increase the likelihood of involvement in risk behaviors (e.g., providing a model for problematic behavior and rising possibilities of involvement in risk behaviors), protective factors diminish the chance of engagement in risk behaviors (e.g., presenting a pattern of prosocial behaviors, social and personal supervision and control, and supportive social environment). In addition, the simultaneous presence of more risk factors and less protective factors increases the likelihood of the adolescent's involvement in problem behaviors (Figure 1) (33, 34).

In the last decades, studies were conducted in various countries to examine the generality of the explanatory model of adolescents' problem behavior based on PBT. The significant merit of such studies is to test the adequacy of an explanatory model, which can be used in different societies (35). An explanatory model's generality across different nations accentuates their dynamic or genotype commonality than phenotypic differences (36). For example, despite a significant difference between the United States and the Republic of China in the social, political, and economic system, Jessor et al. (33) showed that problem behavior is significantly influenced by the same protective and risk factors in both countries and for both genders. Also, Vazsonyi et al. (37) supported the similarities in PBT's explanation of problem behavior in Asian, Eastern, Western European, North American, and Eurasian/Muslim cultures. Thus, the results of these studies make a convincing case for the idea that the explanatory model of PBT has, to a large extent, cross-national generality.

Notwithstanding, there are remarkable differences in culture and educational system between Iran and the countries where PBT has been shown to have generality (specifically Western countries) (38–43). Eastern cultures encourage low arousal emotions, which is in contrast to Western cultures (44); the restriction of emotional expression in Eastern cultures might account for the higher levels of callous-unemotional traits in Asian children compared to children in the United States (45, 46). Additionally, in Iran, the educational system is under the influence of traditional and religious conventions. Iran is one of the rare countries where the Shi'ism stance about ethics like gender segregation is widely used in educational institutions. Further, Islamic values have a considerable impact on adolescents' behavior in Iran. For instance, these norms have limited opportunities to establish relations with the opposite sex (47, 48). In Iran, traditional ethics restrict individuals' independent decision makings, and authority figures like parents, teachers, and elders play an essential role in youngsters' critical decisions (e.g., marrying, buying a house, and choosing a career), so adolescents' behaviors are more influenced by authority figures who encourage them toward prosocial behaviors (48, 49).

On the other hand, in recent years, because of the rapid widening of technology in Iran, most adolescents are getting familiar with Western cultures and imitate them, which is in contrast with their parents' system of beliefs and values. Thus, adolescents experience more conflict with their parents and may experience parental rejection and deprivation from vital support sources. Furthermore, compared to other countries such as the United States and the Republic of China, Iran is a low-income country, and Iranians have been suffering from more severe economic hardship, a factor that increases the rate of crime and delinquent behaviors (50). Financial hardship leads parents to withstand significant distress, and it disrupts the parent-adolescent relationship. As a result, parents lose their control over adolescents' behavior that might increase the risk of adolescents' engagement in problem behaviors (51). Finally, in contrast to individualistic societies such as Western countries, where seeking help is considered a weakness and a disruption to others, in collectivistic nations like Iran, seeking support and expression of feeling are more acceptable (52). Therefore, considering these significant differences in cultural, social, and religious aspects between Iran and other countries, a theory from a different culture cannot be generalized to Iran, and a separate study is needed to test the generality of PBT as an explanation of delinquent behaviors of adolescents in Iran.

Still, to our knowledge, in Iran, no study has employed an integrative theory-based psychosocial model to comprehensively examine problem behaviors among adolescents. Therefore, the current study is designed to examine if the explanatory model for problem behavior involvement (i.e., PBT) accounts similarly for Iranian adolescents' problem behaviors. In this vein, we first explore whether risk factors (i.e., individual vulnerability and opportunity risk availability) in each of the four social contexts (i.e., family, peers, school, and neighborhood) explain delinquent behavior; then, we will examine whether support moderates the influence of risk factors on delinquent behavior. Specifically, based on PBT theory, it is hypothesized that delinquent behaviors would be positively related to individual vulnerability and opportunity risk availability and negatively associated with perceived support.

## Method

### Participants and Procedures

Participants were 392 students aged 13 to 18 years old (*M*_*age*_ = 15.97, *SD* = 1.12; 55.4% girls) in grades 10 (25.5%), 11 (19.1%), 12 (44.9%), and were recruited from four high schools in Tehran (two girl high schools and two boy high schools) through a convenience sampling method. Students, their parents, and teachers were informed regarding the survey administration. Students were surveyed except when they refused to participate or when their parents objected. The administration of the survey was conducted in the classroom on a regular school day. Before beginning the assessment, the students were informed again about the confidentiality of the information and signed the consent form. They were asked to complete the questionnaire in their classroom during a one-hour session under the supervision of a specially trained research assistant (master-level student). Students could ask the supervisor for clarification if they did not understand any question. The participants were also asked to write down their e-mail addresses should they want to receive the result of their completed questionnaire. We distributed the questionnaires to 433 students, and 392 completed questionnaires (response rate of 90%) were recruited. Participants' mothers' education was reported as housekeeper (83.7%) and employed (16.3%); fathers were jobless (1.3%) or employed (98.7%). Before data collection, the present study was evaluated and confirmed by the ethics committee of the Iran University of Medical Sciences (IUMS) (code number: IR.IUMS.REC.1397.1254).

## Measure

### Adolescent Health and Development Questionnaire (AHDQ)

AHDQ (53) is a self-report tool developed based on problem behavior theory. It measures the personality systems, perceived environment systems, and behavior system and structures shaping these systems. AHDQ includes 335 items, which measures problem behaviors, as well as the adolescent's perceptions concerning their relationships with parents, peers, school, neighborhood, and demographic features. The AHDQ includes six domains, but we administered only three of the domains in this study, which include opportunity risk and vulnerability risk (predicting variable), perceived support (moderator variable), and delinquent behaviors (dependent variable). Opportunity risk includes two subscales [i.e., availability (4 items), and gang (2 items)]; vulnerability risk consists of five subscales [i.e., felt stress (3 items), felt depression (3 items), low expectations for success (9 items), low expectations for school achievement (7 items), and low self-esteem (6 items)]; perceived support includes four subscales [i.e., family support (7 items), peers support (4 items), school support (4 items), and neighborhood support (3 items)]; finally, delinquent behaviors includes 10 items. Items scores are summed up to yield the subscales scores.

There are some example for the items of the subscales as follow: availability (*If you wanted some cigarettes to smoke, would you be able to get some at home?*), gang (*Do any of the kids in your neighborhood belong to gangs?*), felt stress (*Is there tension or stress at home in your family?*), felt depression (*In the past six months, have you just felt really down about things?*), low expectations for success (*What are the chances that you will graduate from high school?*), low expectations for school achievement (*How sure are you that you will get at least a B average this year?*), low self-esteem (*What about your ability to do well in school work?*), family support (*Are your parents interested in what you think and how you feel?)*, peers support (*Are your friends interested in what you think and how you feel?*), school support (*Do teachers at your school treat students with respect?*), neighborhood support (*Are people friendly to each other when they meet?*), and delinquent behavior (*During the past six months, how often have you cheated on tests or homework?*).

Mehdizadeh (54) validated the Persian version of the AHDQ with a sample of 1,860 students in Tehran. The results indicated that each of the subscales of individual vulnerability, opportunity risk availability, protective support, and delinquent behaviors domains had almost acceptable internal consistency. The Cronbach's alpha coefficient in both Iranian (54) and US (53) studies were as followed: felt stress (Iran: 0.75, US: 0.76), felt depression (Iran: 0.65, US: 0.86), low expectation for success (Iran: 0.86, US: 0.90), low expectations for school achievement (Iran: 0.72, US: 0.80), low self-esteem (Iran: 0.68, US: 0.65), availability of cigarettes and alcohol in family and neighborhood environment (Iran: 0.86, US: 0.87), opportunity risk gangs (Iran: 0.69, US: 0.81), accessibility to family support (Iran: 0.81, US: 0.86), friends support (Iran: 0.50, US: 0.78), teacher support (Iran.63, US: 0.83), neighborhood support (Iran: 0.66, US: 0.86), and delinquent behavior (Iran: 0.86, US: 0.84).

### Data Analysis

First, in the data screening process, to include as many cases as possible, missing values were examined using the series mean method in SPSS 20; also, we used the Boxplot method to deal with outliers. In the present study, SPSS 20 software was used for data entry and statistical analyses. Descriptive information for variables used in the current study was calculated and presented in Table 1. Data were analyzed using Pearson correlation coefficient and hierarchical multiple regression analysis. For hypothesis testing, we considered *p* = 0.05 as indicating statistically significant results.

**Table 1:** Internal consistency coefficients, means, standard deviations, and pearson correlations among individual vulnerability and opportunity and support with delinquent behaviors.

**Variable**		**Descriptive statistic**				**Correlations**										
		**M**	***SD***	**α**	**MIC**	**1**	**2**	**3**	**4**	**5**	**6**	**7**	**8**	**9**	**10**	**11**
1	Felt stress	8.04	2.55	0.72	0.46											
2	Felt depression	7.60	2.60	0.83	0.45	0.48^**^										
3	Low expectations for success	12.38	4.46	0.61	0.43	0.09	0.25^**^									
4	Low expectations for school achievement	7.96	2.71	0.20	0.36	0.09	0.15^**^	0.38^**^								
5	Low self–esteem	10.88	2.71	0.34	0.18	0.12^*^	0.27^**^	0.32^**^	0.38^**^							
6	Opportunity risk (availability)	6.28	2.42	0.81	0.32	0.14^**^	0.09	0.09	0.16^**^	0.25^**^						
7	Opportunity risk (gangs)	3.49	1.53	0.83	0.47	0.15^**^	0.07	0.01	0.10^*^	0.19^**^	0.36^**^					
8	Family support	21.74	4.25	0.84	0.35	0.36^**^	−0.35^**^	−0.22^**^	−0.22^**^	−0.33^**^	−0.27^**^	−0.22^**^				
9	Friends support	3.81	98.0	0.56	0.24	0.014	0.11^*^	−0.01	−0.13^**^	−0.19^**^	−0.00	−0.07	0.10^*^			
10	Teacher support	10.54	3.09	0.84	0.40	−0.17^**^	−0.14^**^	−0.10^*^	−0.15^**^	−0.23^**^	−0.22^**^	−0.09	0.32^**^	0.12^*^		
11	Neighborhood support	8.07	2.37	0.84	0.47	−0.19^**^	−0.19^**^	−0.07	−0.15^**^	−0.30^**^	−0.20^**^	−0.06	0.31^**^	0.09	0.32^**^	
12	Delinquent behavior	18.64	6.26	0.78	0.28	0.16^**^	0.17^**^	0.21^**^	0.36^**^	0.41^**^	0.51^**^	0.41^**^	−0.39^**^	−0.12^*^	−0.31^**^	−0.26^**^

*M, Mean; SD, Standard Deviation; α, alpha; MIC, Mean Inter-item Correlation*.

** *p < 0.01*;

* *p < 0.05*.

## Results

First, Pearson's correlation were calculated between all study variables. Results indicated that delinquent behavior had a significant positive correlation with individual vulnerability variables consisting of felt stress (*r* = 0.16, *p* < 0.01), felt depression (*r* = 0.17, *p* < 0.01), low expectation for success (*r* = 0.21, *p* < 0.01), low expectations for school achievement (*r* = 0.36, *p* < 0.01), and low self-esteem (*r* = 0.41, *p* < 0.01). Furthermore, delinquent behavior was positively associated with opportunity risk availability variables, including cigarettes and alcohol at home (*r* = 0.51, *p* < 0.01) and access to gangs (*r* = 0.41, *p* < 0.01). Associations between delinquent behaviors and family support (*r* = 0.39, *p* < 0.01), friends support (*r* = 0.12, *p* < 0.05), teacher supports (*r* = 0.31, *p* < 0.01), and neighborhood support (r = 0.26, p < 0.01) were also statistically significant consistent with hypothesized directions (Table 1).

In the present research, our first aim was to test the effect of individual vulnerability (i.e., felt stress, felt depression, low expectation for success, low expectations for school achievement, and low self-esteem) on adolescents' delinquent behaviors. We were also interested in testing whether individual vulnerability interacts with support in its effect on adolescents' delinquent behavior. To test these hypotheses, we used hierarchical multiple linear regression; we entered individual vulnerability variables in the first block, support variables were entered in the second block, and the cross-product variable (individual vulnerability x support) was entered in the third block. The results indicated that, in the first step, individual vulnerability variables explained a statistically significant portion of explained variance (adj *R*^2^ = 0.22, *F*_[5, 385]_ = 22.97, *p* < 0.001). Similarly, in the second step, the support variables resulted in a statistically significant increase in adj *R*^2^ (adj *R*^2^ = 0.29, *F*_[9, 381]_ = 18.40, *p* < 0.001). Finally, in the third step, the cross-product variable (individual vulnerability x support) explained a statistically significant increase in variance explained (adj *R*^2^ = 0.33, *F*_[29, 361]_ = 7.58, *p* = 0.001). Among the analyzed variables, low expectations for school achievement (β = 1.01, *p* < 0.001), low self-esteem (β = 0.75, *p* < 0.01), the interaction between low expectation for success and friends' support (β = −0.55, *p* < 0.02), and interaction between low expectation for success and teacher's support (β = 0.53, *p* < 0.02) had a statistically significant influence on delinquent behaviors (Table 2).

**Table 2:** Results of hierarchical multiple linear regression analyses for individual vulnerability and support and interaction between these variables in their effects on delinquent behaviors (*n* = 392).

**Predictor variables**	**Regression 1**	**Regression 2**	**Regression 3**
Felt stress	0.10 (0.05^*^)		
Felt depression	0.001(0.98)		
Low expectations for success	0.02 (0.73)		
Low expectations for school achievement	0.23 (0.001^**^)		
Low self-esteem	0.31 (0.001^**^)		
Family support		− 0.21 (0.001^**^)	
Friends support		−0.001 (0.93)	
Teacher support		−0.14 (0.001^**^)	
Neighborhood support		−0.05 (0.28)	
Felt stress x Family support protection			−0.02 (0.92)
Felt Depression x Family support protection			0.35 (0.23)
Low expectations for success x Family support protection			−0.10 (0.70)
Low expectations for school achievement x Family support protection			−0.36 (0.19)
Low self-esteem x Family support protection			−0.29 (0.25)
Felt stress x Peer support protection			0.05 (0.81)
Felt depression x Peer support protection			0.05 (0.83)
Low expectations for success x Peer support protection			−0.53 (0.02^*^)
Low expectations for school achievement x Peer support			0.04 (0.83)
Low self-esteem x Peer support protection			−0.30 (0.18)
Felt stress x Teacher support protection			0.17 (0.44)
Felt depression x Teacher support protection			−0.04 (0.85)
Low expectations for success x Teacher support protection			0.55 (0.01^*^)
Low expectations for school achievement x Teacher support protection			−0.30 (0.15)
Low self-esteem x Teacher support protection			−0.30 (0.19)
Felt stress x Neighbors support protection			−0.28 (0.20)
Felt Depression x Neighbors support protection			0.15 (0.49)
Low expectations for success x Neighbors support protection			0.08 (0.67)
Low expectations for school achievement x Neighbors support protection			0.33 (0.14)
Low self-esteem x Neighbors support protection			0.08 (0.73)
R^2^	0.23^**^	0.30^**^	0.39^**^
adj R^2^	0.22^**^	0.29^**^	0.33^**^
F	22.97	18.40	7.94

*β, standardized regression coefficient; SE, standard error*.

** *p < 0.01*;

* *p < 0.05*.

Our second purpose was to determine the effect of opportunity risk availability variables (i.e., opportunity risk availability and opportunity risk gangs) on adolescents' delinquent behaviors and to test whether these variables interact with support in their influence on delinquent behavior. Using hierarchical multiple linear regression, we entered availability variables at the first block, support variables at the second block, and the cross-product variable (availability x support) in the third block. Results of hierarchical multiple linear regression confirmed our hypotheses. In the first step, availability variables explained a statistically significant portion of explained variance (adj *R*^2^ = 0.32, *F*_[2, 388]_ = 91.94, *p* < 0.001). Likewise, in the second step, the support variables increased R^2^, which was statistically significant (adj *R*^2^ = 0.39, *F*_[6, 384]_ = 43.41, *p* < 0.001). In the third step, the cross-product variable (availability x support) explained a statistically significant increase in the variance of delinquent behaviors (adj *R*^2^ = 0.45, *F*_[14, 376]_ = 24.16, *p* = 0.001). Thus, the interaction was statistically significant. Our findings indicated that access to gangs (β = 1.31, *p* < 0.001), the interaction between access to gangs and family support (β = −0.94, *p* < 0.001), opportunity risk availability (β = 0.71, *p* < 0.001), and family support (β = 0.26, *p* < 0.03) have a statistically significant effect on adolescents' delinquent behaviors. Also, the higher beta coefficient of the access to gangs (β = 1.31) indicates that this component is a better predictor of delinquent behaviors than other variables (Table 3).

**Table 3:** Results of hierarchical multiple linear regression analyses for availability and support and interaction between these variables in their effects on delinquent behaviors (*n* = 392).

**Predictor variables**	**Regression 1**	**Regression 2**	**Regression 3**
Opportunity risk availability	0.42 (0.001^**^)		
Opportunity risk gangs	0.26 (0.001^**^)		
Family support		−0.19 (0.001^**^)	
Friends support		−0.06 (0.11)	
Teacher support		−0.11 (0.01^*^)	
Neighborhood support		−0.07 (0.09)	
Opportunity risk availability x Family support			0.01 (0.93)
Opportunity risk gangs x Family support			−0.93 (0.001^**^)
Opportunity risk availability x Friends support			−0.28 (0.19)
Opportunity risk gangs x Friends support			−0.03 (0.86)
Opportunity risk availability x Teacher support			−0.21 (0.24)
Opportunity risk gangs x Teacher support			−0.12 (0.51)
Opportunity risk availability x Neighborhood support			−0.03 (0.88)
Opportunity risk gangs x Neighborhood support			−0.29 (0.14)
R^2^	0.32^**^	0.40^**^	0.47^**^
adj R^2^	0.31	0.39	0.45
F	91.94	43.40	25.76

*β, standardized regression coefficient; SE, standard error*.

** *p < 0.01*;

* *p < 0.05*.

## Discussion

In the current study, we aimed to test the generality of PBT in Iranian culture by examining the relationship between individual vulnerability and opportunity risk availability with delinquent behaviors, considering the moderating role of support.

We found a significant positive relationship between delinquent behavior and individual vulnerability (i.e., felt stress, depression, low expectation for success, low expectations for school achievement, and low self-esteem). Our finding is consistent with previous studies [e.g., (55, 56)]. For instance, Chen et al. (57) found that differences in delinquency between adolescents with community violence exposure experience vs. those without such experiences were smaller when youth reported high levels of future expectations compared to those reporting low levels, implying the critical role of future expectations in youth. Also, consistent with our results denoting the significant effect of low expectation on youth delinquency, an assumption has been proposed by prior researchers, suggesting that proximal factors, such as future or success expectation vs. distal factors, have a more significant influence on resiliency (58, 59). Similarly, to predict delinquent behaviors in youth, some researchers have found that among all of the protective factors, only future expectations interacted with community violence and risk availability, suggesting that the relationship between risk availability and delinquent behaviors is influenced by expectations for the future (57, 60, 61). Also, most studies indicated that youth with a more optimistic view of success and future are less likely to be involved in delinquent behaviors, a hypothesis that has been supported in empirical research (60, 61).

Moreover, our results indicated that high levels of low expectations for success mitigate the positive influence of teacher support on adolescent delinquency. Therefore, teachers fail to have a buffering effect on the relationship between individual vulnerability and delinquency. Also, given increased stress levels stemming from the immense biological and social changes occurring during the adolescence period, the rate of delinquent behaviors increases in adolescents (62). Likewise, adolescents are much more likely to react to environmental influences considering the uncertainty with new expectations and social life (63). Moreover, these findings are consistent with previous work, which has underscored the essential roles of school and family in developing child competency (64, 65). Consistent with prior work [e,g., (66)], our findings document that family support and teacher support are associated with lower levels of delinquent behaviors in adolescents. In the same vein, social control theory establishes a framework for the selection of family, school, and neighborhood contexts to represent protective factors from other contexts. Accordingly, adolescents who are strongly affected by prosocial agents presented by school and parental figures would be more encouraged to stick to conventional behavior, and therefore less likely to engage in delinquency (27). Importantly, during adolescence, individuals become autonomous and begin to individuate (67, 68), so they decrease communication with parents (69). Nonetheless, communication is an essential means for attaining and reinforcing intimacy and closeness, so to satisfy their needs for being independent and to enhance relatedness at the same time, adolescents seek means of communication with those who acknowledge their needs, such as peers, teachers, and neighbors (70). Therefore, when they spend time with other people, especially peers, parents are not available to keep watch over their activities (71). As a result, adolescents may grasp an opportunity to engage in delinquencies (e.g., shoplifting and vandalism) (71, 72).

Additionally, the present study indicated the stronger effects of peer support and low expectation for success on adolescent delinquency than other support variables. Moreover, individual factors such as those associated with vulnerability (e.g., felt stress and low expectation for success) were the main factors leading to a friendship with deviant peers who have problem behaviors (73). Such friendships intensify negative intrapersonal emotions and increase the frequency of delinquent behaviors (74).

Also, in line with previous studies [e.g., (75–78)], our results indicated a significant positive relationship between opportunity risk availability and delinquent behaviors. According to Social Ecology Model (78) and Problem Behavior Theory (79), involvement in gang activities increase the risk of delinquent behaviors, through which gangs provide the opportunity for delinquencies and also recruit youth already at a high risk of problem behaviors (80). Importantly, when interactions between all protective factors and opportunity risk availability were examined simultaneously, our findings support a moderating effect of family support on the relationship between risk availability and youth delinquency. In line with our results, Kliewer et al. (81) indicated that community violence exposure and risk availability were more strongly correlated with externalizing behaviors among youths who reported higher amounts of caregiver emotion regulation (family influence). Conversely, children's emotion regulation and neighborhood influence did not moderate the relationship between risk availability and externalizing behaviors. In the same vein, Kliewer et al. (81) found that taking part in extracurricular activities and positive parent-child relationships decrease the risk effect of risk availability on externalizing behaviors. Some studies [e.g., (82)] found that positive psychosocial and environmental factors can function as protective factors that buffer the adverse effects of low expectations on youth outcomes. Similarly, several studies [e.g., (57, 83, 84)] have supported the buffering effects of protective factors on the relationship between risk availability and problem behaviors, with associations among risk availability and adolescent problem behaviors weakening at a higher level of protective factors, such as family support. Previous research results have shown that if adolescents enjoy emotional support from their parents and have positive and useful experiences in the family (as the primary social institute), they will be secure against risk factors in social environments like the neighborhood and community [e.g., (78)]. Similarly, Keyzers et al. (85) indicated that emotional connections and a good relationship with parents within a secure attachment style are associated with low problem behaviors. Indeed, positive social relationships were reported by adolescents who reflected clear rules and limits in the family, rewards for positive behaviors, parental control over children's behavior, and lack of family conflicts and tensions (86). Specifically, our findings indicated that the association between risk availability and youth delinquency did not vary as a function of protective factors measured at the school, neighborhood, and peer levels, while teacher support was directly associated with lower levels of delinquency. The different figure of results observed for protective factors from different social contexts suggests the inconsistencies may partly be due to cultural differences or the type of protective factor evaluated.

While the findings of the present study can be informative, it should be considered that this study was conducted on high school students who participated in research with their consent. Since this study was done in school, dropped out and fired adolescents, worker youth, imprisoned adolescents, etc., were not considered in the study sample; hence, the obtained results cannot be generalized to all adolescents. In fact, these adolescents may experience a different mechanism of delinquency, which cannot be considered in the study sample of the current study and should be studied in a separate study. Additionally, the self-report method of assessing the adolescent problem behaviors and this method's weaknesses, which in the current research could be the adolescents' sensitivity to some questions concerning delinquency, smoking, and alcohol use, may lead to a lack of honesty in responses given to the questions. Moreover, the other limitation is that due to the low sample size, the analyses were not conducted separately for boys and girls. Despite these limits, the findings of the present research indicated valuable information concerning the relationship between individual vulnerability, support, and delinquent behaviors. The findings of our study can contribute to correct planning and scientific interventions to prevent delinquent behaviors; studies should be done to investigate the longitudinal and qualitative aspects of these plans and interventions. Also, future studies can expand the findings of the present study by considering behavioral brain systems and the brain's arousal levels to find a clearer image of the relationship between individual vulnerability, support, and delinquent behaviors.

## Conclusions

In conclusion, our results suggest that adolescents' involvement in problem behavior in different cultures is independent of where they live and is under the influence of the same individual and social factors (87). Our results add to the sparse literature on problem behavior among adolescents of low industrial and low economic countries like Iran. Also, the significant interactions or moderating effects in this study provide further support for PBT's usefulness and are noteworthy because of the well-known difficulty of demonstrating interaction effects in field studies (88). Furthermore, the results of the present study support the theoretical concepts of PBT and the generality of the PBT explanatory model (89) in Iran. Our results are consistent with previous studies in different cultures such as China, the United States (33), Kenya (90), Hungary, Netherlands, Slovenia, Spain, Switzerland, Taiwan, and Turkey (37), which tested the generality of PBT.

## Author's Note

The information contained in this article was extracted from Mona Darvishi's master's thesis in the School of Behavioral Sciences and Mental Health (Tehran Institute of Psychiatry), Iran University of Medical Sciences, Tehran, Iran.

## Data Availability Statement

The raw data supporting the conclusions of this article will be made available by the authors, without undue reservation.

## Ethics Statement

The studies involving human participants were reviewed and approved by School of Behavioral Sciences and Mental Health (Tehran Institute of Psychiatry), Iran University of Medical Sciences. Written informed consent to participate in this study was provided by the participants' legal guardian/next of kin.

## Author Contributions

MD, MA, ET-C, and MH: conceptualization. MD, ET-C, and MH: methodology. MD and ME: software. MD, ME, and MH: formal analysis. MD and ME: data curation. MD and MH: writing—original draft preparation. MD, ET-C, and MH: visualization, supervision, and project administration. MH: supervision, project administration, resources, and investigation. All authors have read, writing—review and editing, and agreed to the published version of the manuscript.

## Conflict of Interest

The authors declare that the research was conducted in the absence of any commercial or financial relationships that could be construed as a potential conflict of interest.

## Publisher's Note

All claims expressed in this article are solely those of the authors and do not necessarily represent those of their affiliated organizations, or those of the publisher, the editors and the reviewers. Any product that may be evaluated in this article, or claim that may be made by its manufacturer, is not guaranteed or endorsed by the publisher.

## References

[1] Kenchadze E. Delinquent behavior, its characteristics and determining factors. Eur Sci J. (2015) 11. 10.19044/esj.2015.v11n10p%p954484

[2] López CM Andrews Iii AR Chisolm AM de Arellano MA Saunders B Kilpatrick DG. Racial/ethnic differences in trauma exposure and mental health disorders in adolescents. Cultural Diver Ethnic Minority Psychol. (2017) 23:382–7. 10.1037/cdp0000126 27786496 PMC5408300

[3] Gubbels J van der Put CE Assink M. Risk factors for school absenteeism and dropout: a meta-analytic review. J Youth Adolesc. (2019) 48:1637–67. 10.1007/s10964-019-01072-5 31312979 PMC6732159

[4] Jurczyk M Lalak D. Aggressive and delinquent behavior among youth: an empirical study in Poland. Violence and Gender. (2020) 7:188–99. 10.1089/vio.2019.0065

[5] Jokinen T Alexander EC Manikam L Huq T Patil P Benjumea D. A systematic review of household and family alcohol use and adolescent behavioural outcomes in low- and middle-income countries. Child Psychiatr Human Develop. (2020) 52:554–70. 10.1007/s10578-020-01038-w 32785812 PMC8238760

[6] Stenbacka M Moberg T Jokinen J. Adolescent criminality: multiple adverse health outcomes and mortality pattern in Swedish men. BMC Public Health. (2019) 19:400. 10.1186/s12889-019-6662-z 30975117 PMC6460509

[7] Bozzini AB Bauer A Maruyama J Simões R Matijasevich A. Factors associated with risk behaviors in adolescence: a systematic review. Braz J Psychiatry. (2021) 43:210–21. 10.1590/1516-4446-2019-0835 32756805 PMC8023154

[8] Baumer EP Cundiff K Luo L. The contemporary transformation of american youth: an analysis of change in the prevalence of delinquency, 1991–2015. Criminology. (2021) 59:109–36. 10.1111/1745-9125.12264 PMC9910102 36776699

[9] Keyes KM Gary D O'Malley PM Hamilton A Schulenberg J. Recent increases in depressive symptoms among US adolescents: trends from 1991 to 2018. Soc Psychiatry Psychiatr Epidemiol. (2019) 54:987–96. 10.1007/s00127-019-01697-8 30929042 PMC7015269

[10] Andrie EK Tzavara CK Tzavela E Richardson C Greydanus D Tsolia M. Gambling involvement and problem gambling correlates among European adolescents: results from the European Network for Addictive Behavior study. Soc Psychiatry Psychiatr Epidemiol. (2019) 54:1429–41. 10.1007/s00127-019-01706-w 31062040

[11] Chi X Cui X. Externalizing problem behaviors among adolescents in a southern city of China: Gender differences in prevalence and correlates. Child Youth Serv Rev. (2020) 119:105632. 10.1016/j.childyouth.2020.105632

[12] Farrington DP. Conduct Disorder, Aggression, and Delinquency. (2004).

[13] Lerner RM Steinberg L. Handbook of Adolescent Psychology. volume 1: Individual bases of adolescent development: John Wiley & Sons (2009). 10.1002/9780470479193.adlpsy001002

[14] Johnston LD O'Malley PM Bachman JG Schulenberg JE. Monitoring the Future. National Results on Adolescent Drug Use: Overview of Key Findings, 2009. NIH Publication Number 10-7583. National Institute on Drug Abuse (NIDA) (2010). 10.1037/e560352009-001

[15] Bjelopera JP Randol MA editors. The Federal Bureau of Investigation and terrorism investigations. Congressional Research Service, Library of Congress Washington DC, WA (2011).

[16] Esmaielzadeh H Asadi M Miri N Keramatkar M. Prevalence of High Risk Behaviors Among High School Students of Qazvin in 2012. Iran J Epidemiol. (2014) 10:75–82.

[17] Marzban A. Prevalence of high risk behaviors in high school students of Qom, 2016. Pars J Med Sci. (2018) 16:44–51. 10.52547/jmj.16.3.4427531729

[18] Rashid K. Epidemiology of high-risk behaviors among tehran adolescent girls and boys. Soc Welfare. (2015) 15:31–55. 24719706

[19] Lindberg LD Boggess S Williams S. Multiple threats: the co-occurrence of teen health risk-behaviors. In: Trends in the Wellbeing of America's Children and Youth, 1999. Washington, DC: U.S. Department of Health and Human Services (2000). p. 489–504.

[20] Monahan KC Rhew IC Hawkins JD Brown EC. Adolescent pathways to co-occurring problem behavior: The effects of peer delinquency and peer substance use. J Res Adolesc. (2014) 24:630–45. 10.1111/jora.12053 25506186 PMC4260964

[21] Agnew R A A revised strain theory of delinquency*. Social Forces. (1985) 64:151–67. 10.2307/2578977

[22] Brezina T. General Strain Theory. Oxford University Press (2017). 10.1093/acrefore/9780190264079.013.249

[23] Brodie BR. Adolescence and Delinquency: An Object-Relations Theory Approach. Jason Aronson (2007).

[24] Thornberry TP. Toward an interactional theory of delinquency^*^. Criminology. (1987) 25:863–92. 10.1111/j.1745-9125.1987.tb00823.x

[25] Thornberry T. Developmental Theories of Crime and Delinquency. Routledge (2018).

[26] Wiatrowski MD. Social Control Theory and Delinquency. Portland State University (1978).

[27] Hirschi T. A Control Theory of Delinquency: The Craft of Criminology. Routledge (2017). p. 75–90. 10.4324/9781315131511-7

[28] Jensen G. Social Learning Theory and the Explanation of Crime. Taylor & Francis (2017).

[29] Hawkins JD Weis JG. The social development model: an integrated approach to delinquency prevention. J Primary Prevent. (1985) 6:73–97. 10.1007/BF01325432 24271382

[30] Jessor R Jessor SL. Problem Behavior and Psychosocial Development: A Longitudinal Study of Youth. New York, NY: Academic Press (1977).

[31] Donovan JE Jessor R Costa FM. Adolescent health behavior and conventionality-unconventionality: an extension of problem-behavior theory. Health Psychol. (1991) 10:52–61. 10.1037/0278-6133.10.1.52 2026131

[32] Jessor R. Risk behavior in adolescence: a psychosocial framework for understanding and action. J Adolesc Health. (1991) 12:597–605. 10.1016/1054-139X(91)90007-K 1799569

[33] Jessor R Turbin MS Costa FM Dong Q Zhang H Wang C. Adolescent problem behavior in china and the united states: a cross-national study of psychosocial protective factors. J Res Adolesc. (2003) 13:329–60. 10.1111/1532-7795.1303004

[34] Jessor R Graves TD Hanson RC Jessor SL. Problem Behavior Theory: Initial Formulation for the Tri-Ethnic Community Study. The Origins and Development of Problem Behavior Theory: The Collected Works of Richard Jessor. Cham: Springer International Publishing (2016). p. 43–55. 10.1007/978-3-319-40886-6_3

[35] Cai Y Li R Zhu J Na L He Y Redmon P. Personality, perceived environment, and behavior systems related to future smoking intentions among youths: an application of problem-behavior theory in Shanghai, China. PLoS ONE. (2015) 10:e0122276. 10.1371/journal.pone.0122276 25826611 PMC4380425

[36] Lewin K. The conflict between Aristotelian and Galileian modes of thought in contemporary psychology. J Gen Psychol. (1931) 5:141–77. 10.1080/00221309.1931.9918387

[37] Vazsonyi AT Chen P Jenkins DD Burcu E Torrente G Sheu C-J. Jessor's problem behavior theory: cross-national evidence from Hungary, the Netherlands, Slovenia, Spain, Switzerland, Taiwan, Turkey, and the United States. Dev Psychol. (2010) 46:1779–91. 10.1037/a0020682 20873922

[38] Habibi M Tahmasian K Ferrer-Wreder L. Self-efficacy in Persian adolescents: Psychometric properties of a Persian version of the Self-Efficacy Questionnaire for Children (SEQ-C). Int Perspect Psychol. (2014) 3:93–105. 10.1037/a0036059

[39] Ebrahimi A Elhami Athar M Hakim Shooshtari M Karsazi H Storch EA. Psychometric properties of the persian version of the teasing questionnaire 23. Front Psychol. (2021) 12:1021. 10.3389/fpsyg.2021.664736 33935925 PMC8081849

[40] Rezaei O Athar ME Ebrahimi A Jazi EA Karimi S Ataie S. Psychometric properties of the persian version of the inventory of statements about self-injury (ISAS). Borderl Personal Diso Emot Dysregul. (2021) 8:27. 10.1186/s40479-021-00168-4 34772468 PMC8588687

[41] Taheri E Athar ME Ebrahimi A Atashipoor HS Taheri M Mollaee H. The Persian Version of the Personality Beliefs Questionnaire-Short-Form (PBQ-SF): A Psychometric Evaluation. J Rational-Emot Cognit Behav Therapy. (2021). 10.1007/s10942-021-00420-4

[42] Elhami Athar M Ebrahimi A. Psychometric properties and factor structure of the personality inventory for DSM-5–brief form (PID-5-BF) in Iranian student and clinical samples. BMC Psychiatry. (2021). 10.21203/rs.3.rs-440296/v1

[43] Ebrahimi A Elhami Athar M Darvishi M Colins OF. The persian self-report version of the antisocial process screening device (APSD-P): a psychometric evaluation. Front Psychiatry. (2021) 12:1953. 10.3389/fpsyt.2021.760531 34795601 PMC8594756

[44] Lim N. Cultural differences in emotion: differences in emotional arousal level between the East and the West. Integrative Medicine Research. (2016) 5:105–9. 10.1016/j.imr.2016.03.004 28462104 PMC5381435

[45] Fung AL-c Gao Y Raine A. The Utility of the Child and Adolescent Psychopathy Construct in Hong Kong, China. J Clin Child Adolesc Psychol. (2009) 39:134–40. 10.1080/15374410903401138 20390805

[46] Pu W Luo Q Jiang Y Gao Y Ming Q Yao S. Alterations of brain functional architecture associated with psychopathic traits in male adolescents with conduct disorder. Sci Rep. (2017) 7:11349. 10.1038/s41598-017-11775-z 28900210 PMC5595864

[47] Bahador D Somerville AW. Youth at the crossroads: a comparison of American and Iranian adolescence. Adolescence. (1969) 4:1–18.

[48] Moaddel M. The future of Islam after 9/11. Futures. (2004) 36:961–77. 10.1016/j.futures.2004.02.012

[49] Zandpour F Sadri G. Communication in Personal Relationships in Iran: A Comparative Analysis in Communication in Personal Relationships Across Cultures. Thousand Oaks, CA (1996). p. 174–195

[50] Agnew R Matthews SK Bucher J Welcher AN Keyes C. Socioeconomic Status, Economic Problems, and Delinquency. Youth Soc. (2008) 40:159–81. 10.1177/0044118X08318119

[51] Morrison Gutman L McLoyd VC Tokoyawa T. Financial strain, neighborhood stress, parenting behaviors, and adolescent adjustment in urban african american families. J Res Adolesc. (2005) 15:425–49. 10.1111/j.1532-7795.2005.00106.x

[52] Brannan D Biswas-Diener R Mohr CD Mortazavi S Stein N. Friends and family: a cross-cultural investigation of social support and subjective well-being among college students. J Posit Psychol. (2013) 8:65–75. 10.1080/17439760.2012.743573

[53] Jessor R Turbin M Costa F. Survey of Adolescent Health and Development Questionnaire. USA: Cambridge University Press (2004). 10.1037/t82203-000

[54] Mehdizadeh H. Psychometric Properties of the Psychosocial Risk Factors and Protective Problems of Problems in Adolescent. Master's Thesis, Shahid Beheshti University (2015).

[55] Hoffmann JP. Academic underachievement and delinquent behavior. Youth Soc. (2020) 52:728–55. 10.1177/0044118X18767035

[56] Quin D Heerde JA Toumbourou JW. Teacher support within an ecological model of adolescent development: Predictors of school engagement. J Sch Psychol. (2018) 69:1–15. 10.1016/j.jsp.2018.04.003 30558745

[57] Chen P Voisin DR Jacobson KC. Community violence exposure and adolescent delinquency: examining a spectrum of promotive factors. Youth Soc. (2016) 48:33–57. 10.1177/0044118X13475827 33364640 PMC7755159

[58] Bronfenbrenner U Ceci SJ. Nature-nuture reconceptualized in developmental perspective: a bioecological model. Psychol Rev. (1994) 101:568–86. 10.1037/0033-295X.101.4.568 7984707

[59] Rutter M. Resilience in the face of adversity: protective factors and resistance to psychiatric disorder. Br J Psychiatr. (2018) 147:598–611. 10.1192/bjp.147.6.598 3830321

[60] Hong JS Choi J Albdour M Willis TM Kim J Voisin DR. Future Orientation and Adverse Outcomes of Peer Victimization among African American Adolescents. J Aggress Maltreat Trauma. (2021) 30:528–46. 10.1080/10926771.2020.1759747

[61] Stoddard SA Zimmerman MA Bauermeister JA. Thinking about the future as a way to succeed in the present: a longitudinal study of future orientation and violent behaviors among african american youth. Am J Community Psychol. (2011) 48:238–46. 10.1007/s10464-010-9383-0 21104432 PMC3107351

[62] Sigfusdottir ID Kristjansson AL Thorlindsson T Allegrante JP. Stress and adolescent well-being: the need for an interdisciplinary framework. Health Promot Int. (2016) 32:1081–90. 10.1093/heapro/daw038 27153917 PMC5914452

[63] Bacchini D Concetta Miranda M Affuso G. Effects of parental monitoring and exposure to community violence on antisocial behavior and anxiety/depression among adolescents. J Interpers Violence. (2011) 26:269–92. 10.1177/0886260510362879 20234055

[64] Top N Liew J Luo W. Family and school influences on youths' behavioral and academic outcomes: cross-level interactions between parental monitoring and character development curriculum. J Genet Psychol. (2017) 178:108–18. 10.1080/00221325.2017.1279118 28266896

[65] Oldfield J Stevenson A Ortiz E Haley B. Promoting or suppressing resilience to mental health outcomes in at risk young people: The role of parental and peer attachment and school connectedness. J Adolesc. (2018) 64:13–22. 10.1016/j.adolescence.2018.01.002 29408095

[66] Moore GF Cox R Evans RE Hallingberg B Hawkins J Littlecott HJ. School, peer and family relationships and adolescent substance use, subjective wellbeing and mental health symptoms in wales: a cross sectional study. Child Indic Res. (2018) 11:1951–65. 10.1007/s12187-017-9524-1 30524519 PMC6244918

[67] Mahler MS Pine F Bergman A. The Psychological Birth of the Human Infant: Symbiosis and Individuation. Routledge (2018). 10.4324/9780429482915 4445412

[68] Lionetti F Palladino BE Moses Passini C Casonato M Hamzallari O Ranta M. The development of parental monitoring during adolescence: a meta-analysis. Eur J Develop Psychol. (2019) 16:552–80. 10.1080/17405629.2018.1476233

[69] Keijsers L Poulin F. Developmental changes in parent–child communication throughout adolescence. Dev Psychol. (2013) 49:2301–8. 10.1037/a0032217 23477535

[70] Vijayakumar N Pfeifer JH. Self-disclosure during adolescence: exploring the means, targets, and types of personal exchanges. Curr Opin Psychol. (2020) 31:135–40. 10.1016/j.copsyc.2019.08.005 31614251 PMC7130455

[71] Flanagan IML Auty KM Farrington DP. Parental supervision and later offending: a systematic review of longitudinal studies. Aggress Violent Behav. (2019) 47:215–29. 10.1016/j.avb.2019.06.003

[72] Stattin H Kerr M. Parental monitoring: a reinterpretation. Child Dev. (2000) 71:1072–85. 10.1111/1467-8624.00210 11016567

[73] Jessor R. Reflections on six decades of research on adolescent behavior and development. J Youth Adolesc. (2018) 47:473–6. 10.1007/s10964-018-0811-z 29356924

[74] Wang C Hipp JR Butts CT Jose R Lakon CM. Coevolution of adolescent friendship networks and smoking and drinking behaviors with consideration of parental influence. Psychol Addic Behav. (2016) 30:312–24. 10.1037/adb0000163 26962975 PMC11044185

[75] Hsieh H-F Zimmerman MA Bauermeister JA Caldwell CH Xue Y Wang Z. Cumulative risks and promotive factors for Chinese adolescent problem behaviors. J Appl Dev Psychol. (2016) 43:71–82. 10.1016/j.appdev.2016.01.003

[76] Hale DR Viner RM. The correlates and course of multiple health risk behaviour in adolescence. BMC Public Health. (2016) 16:458. 10.1186/s12889-016-3120-z 27246600 PMC4888596

[77] Bouchard M Gallupe O Dawson K Anamali M. No place like home? Availability, opportunity, and substance use in adolescence. J Youth Stud. (2018) 21:747–64. 10.1080/13676261.2017.1420760

[78] Cambron C Kosterman R Catalano RF Guttmannova K Hawkins JD. Neighborhood, family, and peer factors associated with early adolescent smoking and alcohol use. J Youth Adolesc. (2018) 47:369–82. 10.1007/s10964-017-0728-y 28819911 PMC5790639

[79] Jessor R. Problem behavior theory: A half-century of research on adolescent behavior and development. The Developmental Science of Adolescence: History Through Autobiography. New York, NY: Psychology Press (2014). p. 239–256.

[80] Macfarlane A. Gangs and adolescent mental health: a narrative review. J Child Adolesc Trauma. (2019) 12:411–20. 10.1007/s40653-018-0231-y 32318210 PMC7163845

[81] Kliewer W Cunningham JN Diehl R Parrish KA Walker JM Atiyeh C. Violence exposure and adjustment in inner-city youth: child and caregiver emotion regulation skill, caregiver–child relationship quality, and neighborhood cohesion as protective factor. J Clin Child Adolesc Psychol. (2004) 33:477–87. 10.1207/s15374424jccp3303_5 15271605

[82] Brady SS Gorman-Smith D Henry DB Tolan PH. Adaptive coping reduces the impact of community violence exposure on violent behavior among African American and Latino male adolescents. J Abnorm Child Psychol. (2008) 36:105–15. 10.1007/s10802-007-9164-x 17687640 PMC3120137

[83] Ozer EJ Lavi I Douglas L Wolf JP. Protective factors for youth exposed to violence in their communities: a review of family, school, and community moderators. J Clin Child Adolesc Psychol. (2017) 46:353–78. 10.1080/15374416.2015.1046178 26114611

[84] Hardaway CR McLoyd VC Wood D. Exposure to violence and socioemotional adjustment in low-income youth: an examination of protective factors. Am J Community Psychol. (2012) 49:112–26. 10.1007/s10464-011-9440-3 21607826 PMC4071142

[85] Keyzers A Weiler L Haddock S Doty J. Family problem-solving and attachment quality: associations with adolescent risk-taking behavior. J Youth Develop. (2019) 14:70–92. 10.5195/jyd.2019.637

[86] Meldrum RC Connolly GM Flexon J Guerette RT. Parental low self-control, family environments, and juvenile delinquency. Int J Offender Ther Comp Criminol. (2015) 60:1623–44. 10.1177/0306624X15584907 25943365

[87] El-Shenawy OE Shehata A-M. Applying problem behavior theory in a developing Arabic country: Egypt. SAGE Open. (2014) 4:2158244014521819. 10.1177/2158244014521819

[88] McClelland GH Judd CM. Statistical Difficulties of Detecting Interactions and Moderator Effects. US: American Psychological Association (1993). p. 376–390. 10.1037/0033-2909.114.2.376 8416037

[89] Jessor R. Description versus explanation in cross-national research on adolescence. J Adolesc Health. (2008) 43:527–8. 10.1016/j.jadohealth.2008.09.010 19027639

[90] Ndugwa RP Kabiru CW Cleland J. Adolescent problem behavior in nairobi's informal settlements: applying problem behavior theory in sub-Saharan Africa. J Urban Health. (2011) 88:298–317. 10.1007/s11524-010-9462-4 20499192 PMC3132234

